# Promoter methylation inhibits BRD7 expression in human nasopharyngeal carcinoma cells

**DOI:** 10.1186/1471-2407-8-253

**Published:** 2008-09-08

**Authors:** Huaying Liu, Liming Zhang, Zhaoxia Niu, Ming Zhou, Cong Peng, Xiayu Li, Tan Deng, Lei Shi, Yixin Tan, Guiyuan Li

**Affiliations:** 1 Cancer Research Institute, Xiang-Ya School of Medicine, Central South University, Changsha, Hunan, 410078, PR China

## Abstract

**Background:**

Nasopharyngeal carcinoma (NPC) is a head and neck malignancy with high occurrence in South-East Asia and Southern China. Recent findings suggest that epigenetic inactivation of multiple tumor suppressor genes plays an important role in the tumourigenesis of NPC. BRD7 is a NPC-associated bromodomain gene that exhibits a much higher-level of mRNA expression in normal than in NPC biopsies and cell lines. In this study, we explored the role of DNA methylation in regulation of BRD7 transcription.

**Methods:**

The presence of CpG islands within BRD7 promoter was predicted by EMBOSS CpGplot and Softberry CpGFinder, respectively. Nested methylation-specific PCR and RT-PCR were employed to detect the methylation status of BRD7 promoter and the mRNA expression of BRD7 gene in tumor cell lines as well as clinical samples. Electrophoretic mobility shift assays (EMSA) and luciferase assay were used to detect the effects of cytosine methylation on the nuclear protein binding to BRD7 promoter.

**Results:**

We found that DNA methylation suppresses BRD7 expression in NPC cells. In vitro DNA methylation in NPC cells silenced BRD7 promoter activity and inhibited the binding of the nuclear protein (possibly Sp1) to Sp1 binding sites in the BRD7 promoter. In contrast, inhibition of DNA methylation augments induction of endogenous BRD7 mRNA in NPC cells. We also found that methylation frequency of BRD7 promoter is much higher in the tumor and matched blood samples from NPC patients than in the blood samples from normal individuals.

**Conclusion:**

BRD7 promoter demethylation is a prerequisite for high level induction of BRD7 gene expression. DNA methylation of BRD7 promoter might serve as a diagnostic marker in NPC.

## Background

NPC is a head and neck malignancy with high occurrence in South-East Asia and Southern China [1,2]. The development of this EBV-associated cancer may involve cumulative genetic and epigenetic changes in a background of predisposed genetic and environmental factors [3,4]. Genome-wide studies have unraveled multiple chromosomal abnormalities with involvement of specific oncogenes and tumor suppressor genes [5,6]. BRD7 has been recently identified as a bromodomain gene in NPC cells by cDNA Representational Difference Analysis (cDNA RDA) [7]. As a member of the bromodomain genes family, BRD7 may be considered as a component of chromatin remodeling complexes which possess histone acetyltransferase activity [8,9]. Together with E1B-AP5, BRD7 functions as an inhibitor of basic transcription in several viral and cellular promoters in the nucleus [10]. An alternative role of BRD7 arises from the evidence that BRD7 exhibits a much higher level of mRNA expression in normal nasopharyngeal epithelia than in NPC biopsies and cell lines [11,12]. Indeed, over-expression of BRD7 in NPC cells can effectively inhibit cell growth and cell cycle progression from G1 to S phase by transcriptional regulation of some key cell cycle related genes [13-15]. Our previous studies revealed the full-length promoter -404/+46 of BRD7 gene, and showed that Sp1 specifically bound to BRD7 promoter [16]. However, little is known about the down-expression of BRD7 in NPC cells. In this report, we reveal that DNA methylation results in the suppression of BRD7 expression in NPC cells. BRD7 promoter activity is regulated by methylation of CpG sites with the (G+C)-rich promoter region. DNA methylation inhibitor, 5-Aza-CdR, up-regulates BRD7 expression in NPC 5–8F cells. More importantly, the methylation frequency of BRD7 promoter is much higher in the tumor and matched blood samples from NPC patients than that in the blood samples from normal individuals. These results will be helpful in further understanding the transcription-repression mechanism of the BRD7 gene in NPC cells and the establishment of noninvasive approach in the early detection and surveillance of NPC.

## Methods

### Cell culture and antibodies

Most of the cell lines used in this study was from the American Type Culture Collection (ATCC). NPC CNE1, 5–8F (high tumorigenic and metastatic ability) and 6–10B (tumorigenic, but lacking metastatic ability) cell lines were provided by the Cancer Center of Sun Yet-Sen University, (Guangzhou, China). NPC HNE1 cells were provided by Cancer Research Institute of Central South University (Hunan, China). COS7 and BHK-21 cells were cultured in Dulbecco modified Eagle medium (DMEM) supplemented with 10% heat-inactivated fetal bovine serum (FBS), 100 U/ml penicillin and 100 μg/ml streptomycin at 37°C, 5% CO_2_. HNE1, CNE1, 6–10B, 5–8F, SW480 and Hella cells were cultured in RPMI1640 medium containing 10% FBS.

### Bioinformatics

The presence of CpG islands within the upstream region spanning from -1 to -2000 bp of BRD7 gene was analyzed with EMBOSS (European Molecular Biology open software Suite  program CpGplot and Softberry CpGFinder program , respectively.

### Construction of plasmids

pGL3-404/+46 was generated as previously described. pGL3-404/+46/GFP was generated by replacing the luciferase gene of pGL3-enhancer with enhanced green fluorescence protein (EGFP) as follows: EGFP coding region was amplified by PCR using primers 5'-GACTTTCCAAAATGTCGTAACAACTCC-3' (forward) and 5'-GGCTCTAGATTACTTGTACAGCTCGTC-3' (reverse) with pEGFP-C_2_as template, cut with double restriction enzyme NcoI and XbaI, then cloned into vector fragment of pGL3-404/+46 which was cut with the same restriction enzymes NcoI and XbaI to release the luciferase coding region.

### Luciferase assay

Luciferase assay was performed as previously described [16]. Briefly, 4 × 10^5 ^cells were seeded in each well of 12-well plates 24 h prior to transfection, then transfected with 0.5 μg of various BRD7 promoter constructs and 0.25 μg pSV40 β-galactosidase per well by Lipofectamine 2000 Reagent (Invitrogen) according to manufacturer's instruction. Luciferase activity was measured in cell lysates 38 h after transfection using Luciferase Assay kit (Promega). β-galactosidase activity was measured in cell lysates by β-galactosidase Enzyme Assay System (Promega). Experiments were repeated at least three times with three replicates per sample. Results were normalized against β-galactosidase activity.

### Direct GFP fluorescence assay

4 × 10^5 ^cells were seeded in each well of 12-well plates 24 h prior to transfection. Next day, every well was transfected with 0.5 μg of pGL3/-404,+46/EGFP or CH_3_-pGL3/-404,+46/EGFP in by using Lipofectamine 2000 Reagent according to manufacturer's instruction. EGFP fluorescence was observed 38 h after transfection using an AX-80 analytical microscope system (Olympus, Tokyo, Japan).

### RT-PCR

RT-PCR was performed as previously described [16]. The single-stranded cDNA was amplified by using primers as follows: BRD7 primer (forward) 5'-CAAGCTCTTTAGCCAAACAAGAA-3', (reverse) 5'-TCATTCCTGAGTGCAACAGC-3'; GAPDH primer (forward) 5'-TCTAGACGGCAGGTCAGGTCCACC-3', (reverse) 5'-CCACCCATGGCAAATTCCATGGCA-3'. PCR was carried out for 28 cycles using a step cycle of 94°C for 40 s, 58°C for 40 s, 72°C for 1 min, followed by 72°C for 10 min. GAPDH primer was added to the reactions at the end of the fifth cycle.

### Nested methylation-specific PCR analyses

The DNA methylation status was established by PCR analysis of bisulfite-modified genomic DNA, which induces chemical conversion of unmethylated, but not methylated, cytosine to uracil, using two procedures. First, methylation status was analyzed by bisulfite genomic sequencing of both strands of the corresponding CpG islands. The second analysis used methylation-specific PCR using primers specific for either the methylated or modified unmethylated DNA. Methylation-specific primer (forward): 5'-AGTTTGAGCGGTGGATTTCGTTTC-3', (reverse) 5'-GGTTCGGTCGGATATGGGTAAGAAG-3'; Unmethylation-specific primer (forward): 5'-AAAGATGAGAGTTTGAGTGGTGGATTTT-3', (reverse) 5'-GGGGTTTGGTTGGATATGGGTAAGAAG-3'.

### Sodium bisulfite modification and genomic sequencing

Genomic DNA was extracted from blood or cultured cells with or without a 72 h pretreatment with 5-Aza-CdR, using the DNA-easy kit (Qiagen) according to the manufacturer's instructions. Two μg of DNA was denatured in 50 μl of 0.3 M NaOH for 15 min at 37°C. For the chemical modification of DNA, 520 μl of 3 M sodium bisulfite (Sigma) and 30 μl of 10 mM hydroquinone (Sigma) were added to the DNA solution and the samples were mixed, overlaid with mineral oil, and incubated at 50°C overnight. Modified DNA was purified with the Wizard DNA Clean-up system (Promega) and eluted in water. As a final step, NaOH was added to a final concentration of 0.3 M, and the samples were incubated for 5 min at room temperature. DNA was precipitated by ethanol and resuspended in water. The sequence of interest in the bisulfite-reacted DNA was PCR-amplified in a reaction mixture containing dNTPs, PCR buffer, Taq enzyme, and primers. For each reaction, 1 μl (~50 ng) of bisulfited DNA was used in 25 μl reaction volume. DNA fragments were gel-purified with the QIAquick Gel Extraction kit (Qiagen) cloned into pGEM/T-easy vector (Invitrogen). Clones with appropriate sized inserts were sequenced.

### In vitro DNA methylation and transient transfection

The methylated plasmids (Met-pGL3/-404,+46 and Met-pGL3/-404,+46/GFP) were generated by incubating 40 μg of plasmid DNA (pGL3/-404,+46 and pGL3/-404,+46/GFP) with 100 units SssI methylase in reaction buffer consisting of 50 mM NaCl, 10 mM Tris-HCl, 10 mM MgCl_2_, 1 mM dithiothreitol, pH 7.9, and 160 μM S-adenosylmethionine according to the manufacturer's instructions (New England Biolabs, Inc.). Reactions were carried out at 37°C overnight. Complete methylation was verified by digestion with the methylation-sensitive restriction enzyme HpaII. Only plasmids that showed a complete protection from HpaII digestion were used in the transfection experiments. The methylated plasmid DNA was purified by the Wizard DNA Clean-up system (Promega) and transfected into COS7 and 5–8F cells in parallel with the unmethylated pGL3/-404,+46 and pGL3/-404,+46/GFP, respectively. Luciferase activity was analyzed at 38 h after transfection.

### Electrophoretic mobility shift assays (EMSA)

Nuclear extracts were prepared, quantified, and used for EMSA with double strand probes or competitors as described previously [16]. The methylated -353/-337 and -330/-317 oligonucleotide were prepared by incubating 20 μg of unmethylated -353/-337 (Sense: 5'-GATCCCGCCCCGGCCCCGCCCTCGG-3', anti-sense:5'-CCGAGGGCGGGGCCGGGGCGGGATC-3') and -330/-317 (Sense: 5'-CGGCCCCGCCCCCGGCCCGCGAGCT-3', anti-sense: 5'-AGCTCGCGGGCCGGGGGCGGGGCCG-3') with 80 units of SssI CpG methylase at 37°C overnight. The reaction mixture was then heated at 65°C to inactivate the methylase, purified by polyacrylamide gel electrophoresis, and concentrated with Centricon 3 microconcentrators. Nuclear extracts were incubated for 20 min on ice in the presence or absence of unlabeled competitor oligonucleotides followed by the addition of the end-labeled probe and 15 min incubation on ice.

### 5-Aza-CdR and TSA treatment

For the 5-Aza-CdR treatment, DNA methyltransferase inhibitor, 5-Aza-CdR, was added to 2 × 10^6 ^cells at final concentrations from 1.875 to 15 μM for 72 h. For trichostatin A (TSA) treatment alone, deacetylase inhibitor TSA was added to 2 × 10^6 ^cells at final concentrations from 150 to 5000 nM for 48 h. For the treatment of 5-Aza-CdR combined with TSA, 1000 nM of TSA was added to 2 × 10^6 ^cells for 48 h at the end of the treatment of 3.75 μM 5-Aza-CdR.

### Tumor and blood samples

All samples were collected from the Xiangya Hospital of Central South University and the Hunan Tumor Hospital, Changsha, Hunan, China. All patients were diagnosed by pathological examination. Totally 18 NPC patients and 16 normal individuals were used in this study. Written informed consent was obtained from all studied participants. The study was approved by the ethical review committees of the appropriate institutions. Five-to-10 ml peripheral blood samples were taken from each individual.

## Results

### A CpG island is overlapped with BRD7 promoter

**Figure 1:**
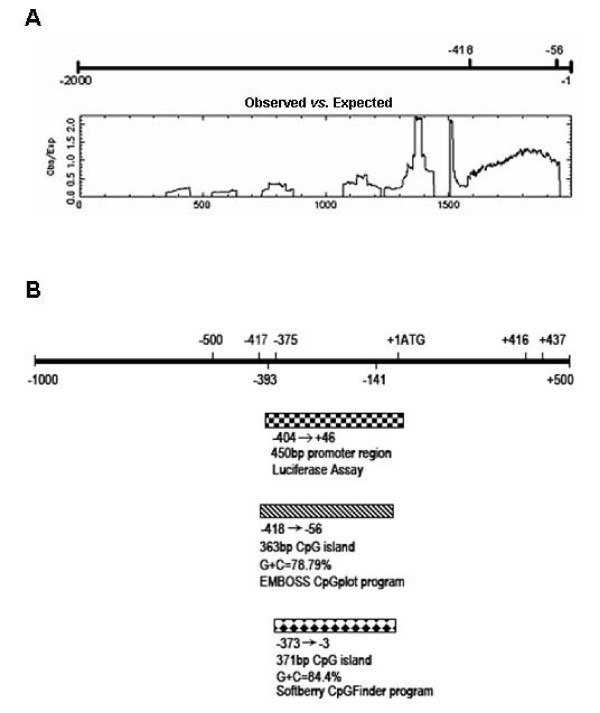
**Overlapping of CpG island with BRD7 promoter.** (A) A CpG island spanning from -481 to -56 bp upstream of BRD7 gene identified by EMBOSS CpGplot program. (B) Schematic representation of BRD7 promoter -404/+46 (marked with the square) and the CpG island in the upstream sequence of BRD7 gene. The CpG island (hatched box) of BRD7 gene was predicted by EMBOSS CpGplot program whereas the CpG island (diamond-shaped box) of BRD7 gene was predicted by the Softberry CpGFinder program. The translation start site is position +1 and the rest of the sequence is numbered relative to it.

After depositing 2000-bp of the upstream gDNA sequence of BRD7 gene, a CpG island spanning from -418 to -56-bp was identified by using EMBOSS software (Fig. 1A, B), whereas a CpG island spanning from -374 to -4-bp was identified by using Softberry CpG Finder Program (Fig. 1B). The sequences of the CpG islands predicted by these two programs overlapped with each other, as well as with the sequences of BRD7 promoter (Fig. 1B). The overlapping region was a 317-bp-long sequence (-373 to -56 bp).

### Down-regulation of BRD7 gene expression in NPC cells is due to partly methylation of BRD7 promoter

**Figure 2:**
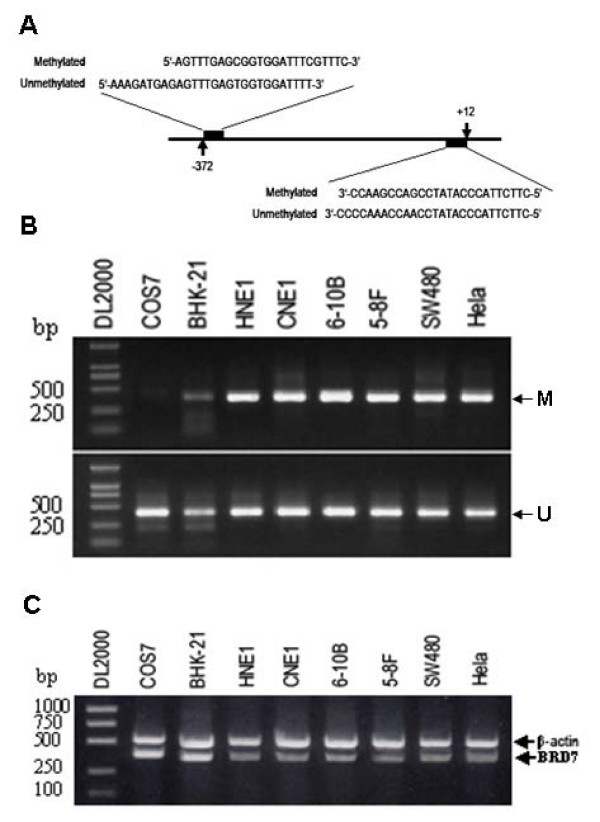
**Down- regulation of BRD7 in NPC cells is due to partial methylation of BRD7 promoter.** (A) Map of the methylation- and unmethylation-specific primer in BRD7 promoter region. (B) PCR amplification of BRD7 promoter region by using methylation- and unmethylation-specific primer. PCR product was size-fractionated on agarose gels and bands visualized by ethidium-bromide staining of the gels. The presence of a PCR band in the lane "M" indicates methylated genes, while the presence of a PCR band in the lane "U" indicates unmethylated genes. (C) Detection of BRD7 mRNA expression in the cell lines by RT-PCR.

Previous studies have shown that BRD7 was down-regulated in NPC biopsies and NPC cell lines [11]. Genomic DNA obtained from various cell lines was treated with sodium bisulfite under conditions where cytosines are converted to uracils, while methylated cytosines remain unmodified. By using methylation-specific and unmethylation-specific primers described in Fig. 2A, we performed methylation-specific PCR and revealed that HNE1, CNE1, 6–10B, 5–8F, SW480 and Hela cells exhibited a methylated BRD7 promoter, whereas no methylation of BRD7 promoter was found in COS7 and BHK-21 cells (Fig. 2B). RT-PCR showed down-expression of BRD7 mRNA in HNE1, CNE1, 6–10B, 5–8F, SW480 and Hela cells as compared to COS7 and BHK-21 cells. The data indicated that the mRNA expression of BRD7 gene is inversely correlated to the methylation status of BRD7 promoter in NPC cell lines.

### DNA methylation inhibitors 5-Aza-CdR augments endogenous mRNA and reverses the methylation status of BRD7 promoter in NPC cells

**Figure 3:**
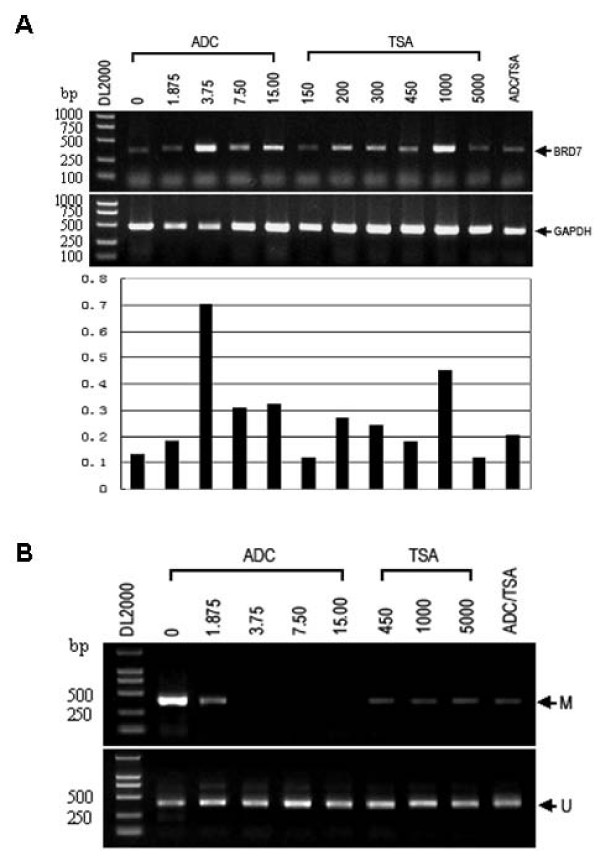
**DNA methylation inhibitors 5-Aza-CdR augments endogenous mRNA expression of BRD7 gene and reverses the methylation status of BRD7 promoter.** (A) RT-PCR amplification of BRD7 expression in 5–8F cells treated with 0–15 μM 5-Aza-CdR, 150–450 nM TSA, and 7.5 μM 5-Aza-CdR combined with 300 nM TSA. (B) PCR amplification of BRD7 promoter region with methylation- and unmethylation-specific primer by using modified gDNA from 5–8F cells in the presence of vehicle or indicated concentration of 5-Aza-CdR, TSA or 5-Aza-CdR combined with TSA. The presence of a PCR band in the lane "M" indicates methylated promoter fragment, while the presence of a PCR band in the lane "U" indicates unmethylated promoter fragment.

To determine whether DNA methylation and chromatin modification contribute to the regulation of BRD7 expression in NPC 5–8F cells, BRD7 mRNA level was measured in the presence of various concentrations of 5-Aza-CdR alone, TSA alone or 5-Aza-CdR combined with TSA. As shown in Fig. 3A, BRD7 mRNA expression in NPC 5–8F cells was increased by 7 fold by 5-Aza-CdR (3.75 μM) and by 4 fold by TSA (1000 nM) relative to controls. The addition of TSA to 5-Aza-CdR did not result in additional enhancement of the BRD7 gene expression in NPC 5–8F cells. Methylation-specific PCR results showed that as low as 3.75 μM of 5-Aza-CdR was sufficient to completely reverse the methylation of BRD7 promoter in 5–8F cells (Fig. 3B). These data suggest that hypomethylation increased BRD7 mRNA expression in 5–8F cells. Therefore, the restoration of BRD7 induction by 5-Aza-CdR or TSA treatment could be related a direct demethylation of BRD7 promoter.

### Bisulfite treatment and sequencing analysis identifies methylated cytosines in BRD7 promoter

**Figure 4:**
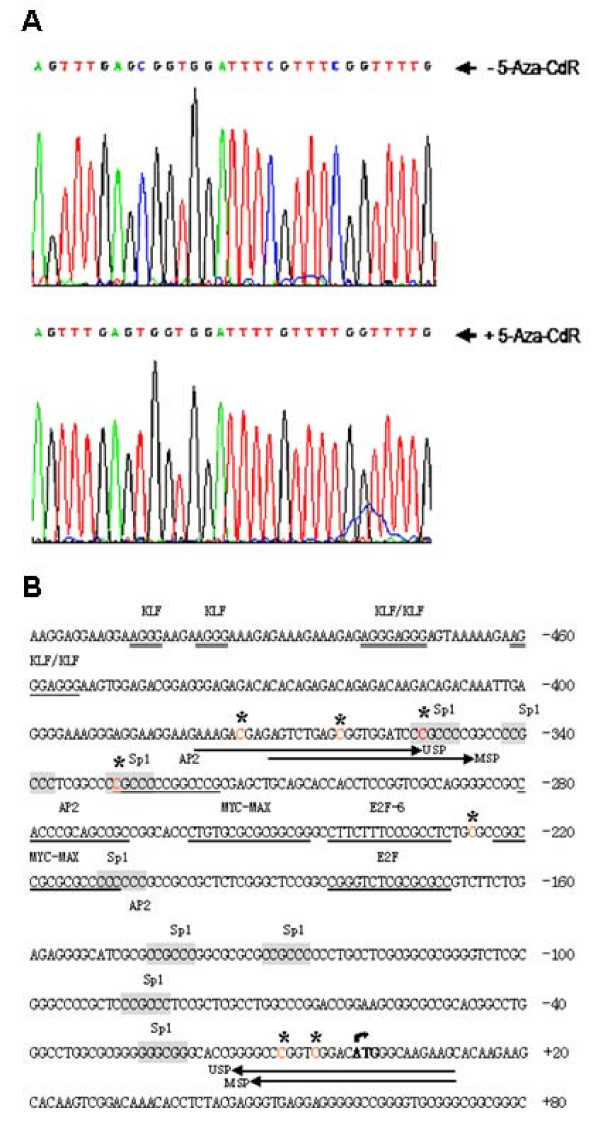
**Bisulfite treatment and sequencing analysis identifies methylation of BRD7 promoter.** (A) Representative sequencing graphs of BRD7 promoter region in 5–8F cells treated with or without 5-Aza-CdR. Modified and unmodified cytosines are indicated by *arrows*. (B) Schematic depiction of methylated cytosine in BRD7 promoter region. The Cytosine under the "*" in the dinucleotide CpG indicates methylated cytosines.

Sodium bisulfite deaminates unmethylated cytosine to uracil in single-stranded DNA under conditions in which the 5-methylcytosine remains nonreactive. Thus, all cytosine residues remaining at the time of sequencing represent cytosines that were methylated in the original DNA sequence. Genomic DNA from 5–8F cells treated with or without 3.75 μM of 5-Aza-CdR was analyzed. Sequencing analysis showed that the cytosines (labeled with *) at -374, -362, -352, -329, -226, -9, -5 bp were methylated in BRD7 promoter of 5–8F cells and were unmethylated in 5–8F cells treated with 3.75 μM of 5-Aza-CdR (Fig. 4B). Two "C" (labeled with *) at -260 and -170 bp appear. It could not be due to CpG methylation, possibly due to insufficient bisulfited treatment.

### Cytosine methylation inhibits nuclear protein binding to BRD7 promoter

**Figure 5:**
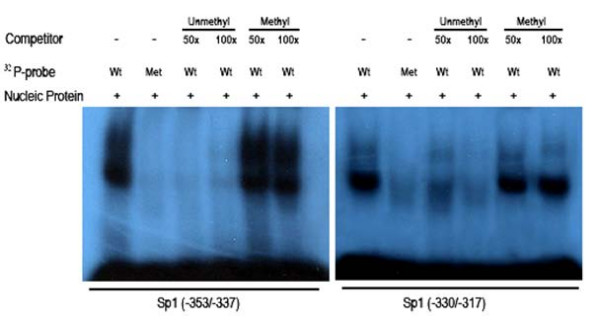
**Cytosine methylation inhibits transcription factor binding to its corresponding binding sites in BRD7 promoter.***Left panel: *Comparison of the nuclear binding capabilities of unmethylated and methylated -353/-337 probes by EMSA. Results are representative of three independent experiments. Excess amounts of unlabeled unmethylated -353/-337, methylated -353/-337 were added as competitors. *Right panel: *Comparison of the nuclear binding capabilities of unmethylated and methylated -330/-317 probes by EMSA. Excess amounts of cold unmethylated -330/-317, methylated -330/-317 were added as competitors. NP: nuclear protein; Wt: wild type probe; Met: methylated probe; CH_3_-P: SssI methylase treated probe.

Cytosine methylation in the promoter region, when present within regulatory elements, could potentially interfere with binding of specific transcription factors to these motifs. Our previous studies confirmed that the MYC-MAX binding site at -260/-246 was non-specific [16]. To investigate whether cytosine methylation within Sp1 binding sites at -353/-337 and -330/-317 interfere with nuclear factor binding, we compared the binding abilities in EMSA reactions of a 20-bp oligomer (nucleotides -353/-337 and -330/-317), which contained the two different Sp1 elements and neighboring cytosines, in unmethylated and methylated forms. First we examined the abilities of unmethylated and methylated -353/-337 to compete with the unmethylated -353/-337 probe in binding to nuclear proteins from 5–8F cells. As seen in Fig. 5A, two sequence-specific gel shift complexes were observed with labeled unmethylated -353/-337 as a probe (lane 1 of Fig. 5), but no complex was formed with labeled methylated -353/-337 as a probe (lane 2 of Fig. 5). In competition EMSA reactions, 50-fold excess of unlabeled unmethylated -353/-337 oligomers were sufficient to completely inhibit complex formation (lane 3, 4 of Fig. 5), but none of the DNA-protein bands were inhibited by the addition of a 100-fold excess of unlabeled methylated -353/-337 oligomers (lane 5, 6 of Fig. 5), suggesting that the unmethylated -353/-337 oligomer binds activated protein. Then we compared the abilities of unmethylated and methylated -330/-317 oligomers to bind nuclear proteins from 5–8F cells in EMSA reactions using labeled unmethylated -330/-317 or methylated -330/-317 as probes. A strong and a weak DNA-protein complexes were formed with labeled unmethylated -330/-317 as probes (lane 7 of Fig. 5), whereas no DNA-protein complexes were formed with methylated -330/-317 as probes (lane 8 of Fig. 5). In the competition assay, both of them were completely inhibited with cold unmethylated -330/-317 (lane 9 of Fig. 5), but none of them were inhibited by cold methylated -330/-337 (lane 11, 12 of Fig. 5). These results suggested that methylation of cytosines at -353/-337 and -330/-317 significantly inhibited nuclear protein (possibly Sp1) binding to BRD7 promoter.

### In vitro cytosine methylation of BRD7 promoter silence its activity

**Figure 6:**
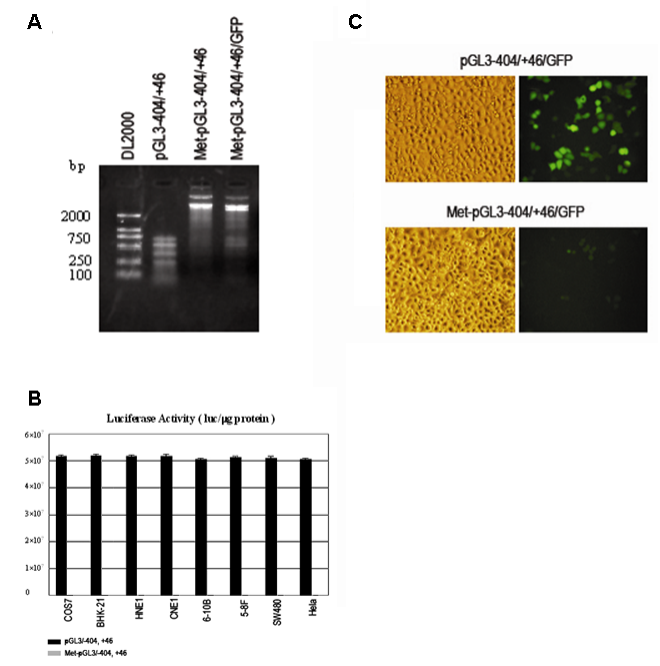
**In vitro cytosine methylation of BRD7 promoter silence its activity in NPC cells.** (A) Detection of the methylation effects of Met-pGL3-404/+46 and Met-pGL3-404/+46/GFP by restriction enzyme cutting of HpaII. pGL3-404/+46 was used as a control. (B) Detection of the luciferase activity of methylated BRD7 promoter construct Met-pGL3/-404,+46 in COS7, BHK-21, HNE1, CNE1, 6–10B, 5–8F, SW480, and Hela cells. Luciferase activity in COS7 and 5–8F cells is represented by black and gray histograms, respectively. All of the constructs were cotransfected with the SV40 β-galactosidase vector for normalizing transfection efficiency. Data are the means ± S.D. of three independent experiments. (C) Detection of the luciferase activity of modified GFP reporter construct Met-pGL3/-404,+46/EGFP in 5–8F cells. The full-length modified promoter construct pGL3/-404,+46/EGFP was used as a positive control. 38 h after transfection, the signal of EGFP fluorescence driven by promoter fragment -404/+46 or -266/-212 was observed by using an AX-80 analytical microscope system (Olympus, Tokyo, Japan).

To investigate the effect of in vitro CpG methylation on BRD7 promoter activity, we treated BRD7 promoter reporter construct pGL3-404/+46, pGL3-404/+46/GFP with SssI methylase. After confirming the complete methylation status with restriction enzyme HpaII (Fig. 6A), Met-pGL3-404/+46, was transfected into COS7, BHK-21, HNE1, CNE1, 6–10B, 5–8F, SW480 and Hela cells. As shown in Fig. 6B, the luciferase activity driven by methylated BRD7 promoter was significantly decreased in all the detected cell lines. To further confirm the effects of methylation on promoter activity, methylated GFP reporter construct Met-pGL3-404/+46/GFP was transfected into 5–8F cells. It exhibited no GFP fluorescence in 5–8F cell lines (Fig. 6C). These results were consistent with that of the luciferase assay, indicating that DNA methylation of BRD7 promoter completely silenced its activity.

### Frequent aberrant methylation of BRD7 promoter in NPC patients

**Figure 7:**
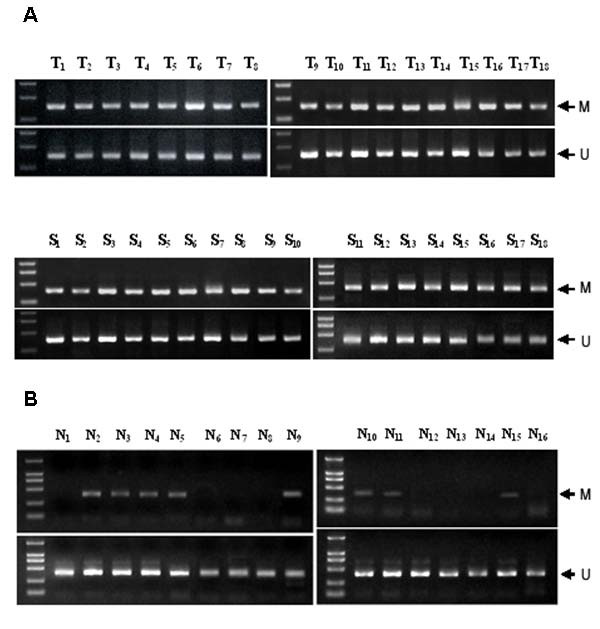
**Detection of methylation frequency of BRD7 promoter in tumor biopsies and blood samples from NPC patients (A) as well as blood samples from corresponding normal individuals (B) by methylation-specific PCR.** T: tumor biopsies, B: blood samples, N: blood samples from normal individuals, M: amplified product with primer recognizing methylated sequences, U: amplified product with primer recognizing unmethylated sequences.

We examined the methylation status of BRD7 promoter in paired tumor biopsies and blood samples from NPC patients and from normal individuals by using a methylation-specific PCR. Aberrant promoter methylation of BRD7 gene was detected in 18 of 18 (100%) tumor biopsies (Top of Fig. 7A) and 18 of 18 (100%) (Bottom of Fig. 7A) matched blood samples of NPC patients, respectively. In contrast, very weak promoter methylation of BRD7 gene was observed in 8 blood samples of 16 normal, healthy, age-matched controls (Fig. 7B).

## Discussion

BRD7 is a recently identified bromodomain gene. It exhibits much higher-level of mRNA expression in normal nasopharyngeal epithelia than in NPC biopsies and cell lines [11,12]. Over-expression of BRD7 in NPC cells is effective in inhibiting cell growth and cell cycle progression of NPC cells [13-15], but little is known about its down-expression in NPC cells. In this study, we found that BRD7 promoter is hemimethylated in a number of NPC cell lines including HNE1, CNE1, 6–10B and 5–8F cell lines, and that the methylation status of BRD7 promoter is inversely proportional with BRD7 mRNA expression in NPC cells. Thus, pharmacological inhibition of DNA methylation by 5-Aza-CdR enhanced BRD7 mRNA expression in NPC cells. This is in agreement with previous studies that, indeed, hemimethylation is sufficient to inhibit the expression of p16ink4A [17] and hMLH1 gene [18] in HCT116 and HT29 cell lines, respectively. Numerous studies have suggested that DNA methylation can suppress gene transcription either by directly inhibiting the interaction of transcription factors with their regulatory sequences or by attracting methylated DNA binding proteins that, in turn, recruit histone deacetylases and histone methyltransferases, resulting in an inactive chromatin structure [19,20]. Our study indicates that DNA methylation represses BRD7 gene transcription by directly inhibiting the interaction of transcription factors with their regulatory elements, as judged by the inability of TSA to potentiate 5-Aza-CdR-mediated expression of BRD7 gene. Sp1 is a well-investigated factor that regulates transcription through specific sequences in G/C-rich promoter regions and is often critical for transcription initiation of TATA-less promoters [21]. We identified several Sp1 binding sites in BRD7 promoter. Sp1 has high affinity to BRD7 promoter [16]. Sequence analysis of the bisulfite-modified BRD7 promoter demonstrated that cytosine residues flanking functional Sp1 elements at -353/-337 and -330/-317 are methylated. It is known that methylation of specific cytosine residues in or near transcription regulatory motifs can block accessibility of the transcription factor [22-24]. Indeed, we found that methylation of cytosines flanking the -353/-337 and -330/-317 element impaired the ability of nuclear protein to bind the Sp1 binding sites in BRD7 promoter. Moreover, in vitro methylation of BRD7 promoter construct with SssI methylase leads to an almost complete loss of the activity of BRD7 promoter in NPC cell lines. NPC is highly radiosensitive and chemosensitive, but treatment of patients with locoregionally advanced disease remains problematic [25,26]. New biomarkers for NPC, including DNA copy number of EBV or methylation of multiple tumour suppressor genes, which can be detected in serum and nasopharyngeal brushings, have been developed for the molecular diagnosis of this tumor. Recent findings suggest that epigenetic inactivation of multiple tumor suppressor genes plays an important role in the tumourigenesis of NPC, such as aberrant methylation of the 5-CpG island of Ras association domain family 1A (RASSF1A), RARβ2, death-associated protein kinase (DAP-kinase), p16 (CDKN2A), p15 (CDKN2B), p14 (ARF) and O6-methylguanine DNA methyltransferase (MGMT), DLC1, TSLC1, TIG1 in NPC [27-33]. In the present study, among the 18 NPC patients, aberrant promoter methylation of BRD7 gene was detected in 100% of tumor biopsies and matched blood samples of NPC patients. In contrast, weak promoter methylation of BRD7 gene was observed in half of the blood samples from normal, healthy, age-matched individuals, indicating that epigenetic inactivation of BRD7 gene plays an important role in the tumorigenesis of NPC. This is a provocative observation, suggesting that the methylation status of BRD7 promoter may serve as a clinical biomarker for early detection and prescreening patients with clinical symptoms or individuals at high risk as well as in monitoring patients for recurrence. Further studies are necessary to confirm this.

## Conclusion

BRD7 promoter demethylation is a prerequisite for high level induction of BRD7 gene expression. DNA methylation of BRD7 promoter might serve as a diagnostic marker in NPC.

## Competing interests

The authors declare that they have no competing interests.

## Authors' contributions

HL participated in the study design, drafted the manuscript and carried out the molecular genetic studies. LZ performed the molecular biology studies. ZN carried out the cell biology studies. MZ was responsible for the study coordination. CP helped to carry out the molecular biology studies. XL helped to carry out the cell biology studies. TD performed the bioinformatics studies. LS participated in collecting fresh blood samples. YT helped to collect blood samples. GL participated in the study design and assessed the data integrity. All authors helped to draft the manuscript, and to read and approve the final version.

## Pre-publication history

The pre-publication history for this paper can be accessed here:


